# Temporal and spatial dynamics of competitive parapatry in chewing lice

**DOI:** 10.1002/ece3.5183

**Published:** 2019-06-14

**Authors:** David J. Hafner, Mark S. Hafner, Theresa A. Spradling, Jessica E. Light, James W. Demastes

**Affiliations:** ^1^ Museum of Southwestern Biology University of New Mexico Albuquerque New Mexico; ^2^ Museum of Natural Science and Department of Biological Sciences Louisiana State University Baton Rouge Louisiana; ^3^ Department of Biology University of Northern Iowa Cedar Falls Iowa; ^4^ Department of Wildlife and Fisheries Sciences Texas A&M University College Station Texas

**Keywords:** chewing lice, contact zones, dispersal distance, hybrid zones, pocket gophers, species replacement

## Abstract

We synthesize observations from 1979 to 2016 of a contact zone involving two subspecies of pocket gophers (*Thomomys bottae connectens* and *T. b. opulentus*) and their respective chewing lice (*Geomydoecus aurei* and *G. centralis*) along the Río Grande Valley in New Mexico, U.S.A., to test predictions about the dynamics of the zone. Historically, the natural flood cycle of the Rio Grande prevented contact between the two subspecies of pocket gophers. Flood control measures completed in the 1930s permitted contact, thus establishing the hybrid zone between the pocket gophers and the contact zone between their lice (without hybridization). Since that time, the pocket gopher hybrid zone has stabilized, whereas the northern chewing louse species has replaced the southern louse species at a consistent rate of ~150 m/year. The 0.2–0.8 width of the replacement zone has remained constant, reflecting the constant rate of chewing louse species turnover on a single gopher and within a local pocket gopher population. In contrast, the full width of the replacement zone (northernmost *G. centralis* to southernmost *G. aurei*) has increased annually. By employing a variety of metrics of the species replacement zone, we are better able to understand the dynamics of interactions between and among the chewing lice and their pocket gopher hosts. This research provides an opportunity to observe active species replacement and resulting distributional shifts in a parasitic organism in its natural setting.

## 1 INTRODUCTION

Most studies of species contact are restricted to two taxa at a single point in time, despite theoretical studies indicating the value of considering interactions among multiple species (Svenning et al., 2014) over long time periods (Buggs, 2007). In particular, studies of contact zones at a single point in time contribute to the general assumption that contact between contiguously distributed species is relatively stable geographically (witness countless maps of species distributions in field guides). Certainly, the impact of long‐term climate change on species distributions (e.g., during climate oscillations of the Quaternary) is well understood and appreciated. But major distributional changes resulting from climate change can occur over far shorter periods (decades, even years; Frey, 1992), and rapid distributional expansions or reductions (including extirpation or extinction) can result from environmental perturbations that create or eliminate dispersal corridors and barriers (e.g., bridges, dams, river diversion, deforestation, or interference with natural fire cycles). Because such changes occur rapidly, opportunities to study them while they are actively occurring are rare, yet have the potential to provide insight into the dynamics of species distributions. Study of species at the margins of their geographic distributions may reveal factors critical to their ecological limitations or other life‐history parameters (Hall, 1946; Hargreaves & Eckert, 2014). In a similar sense, study of the interactions of species in zones of contact has the potential to reveal emergent features of their natural histories that could not be discovered by separate studies of each species in isolation.

Contact zones involving host species and their obligate parasites are a special case of multiple‐species contact zones, in that the dynamics of the parasite species’ contact zone and transmission of parasites to alternate hosts are determined by specializations and behaviors of both the parasites and their hosts. The contact between the host species is generally viewed as reflecting the current distribution of their respective habitats, whether along a wide front or in patchy islands of habitat. These habitats shift over ecological and geological time. In contrast, from the perspective of the parasite species, the hosts are patches of habitat that move constantly both in daily activity and in annual dispersal. Thus to understand the dynamics of the parasite contact zone, it is important to consider the basic life history and dynamics of both the parasite and the host.

### 1.1 Pocket gophers, chewing lice, and history of the San Acacia contact zone

Pocket gophers of the genus *Thomomys* (Rodentia: Geomyidae) are fossorial, solitary, and aggressively territorial, and encounters among individuals, except when mating, are generally avoided. Individuals probably live from 1 to 3 years, with high juvenile mortality during above‐ground dispersal following birth in the spring (Hafner, 2016). Based on the few published measures of dispersal distance in pocket gophers, average annual dispersal distance is 62–117 m/year, and maximum dispersal distance is 122–300 m (Daly & Patton, 1990; Howard & Childs, 1959; Vaughan, 1963).

**Figure 1:**
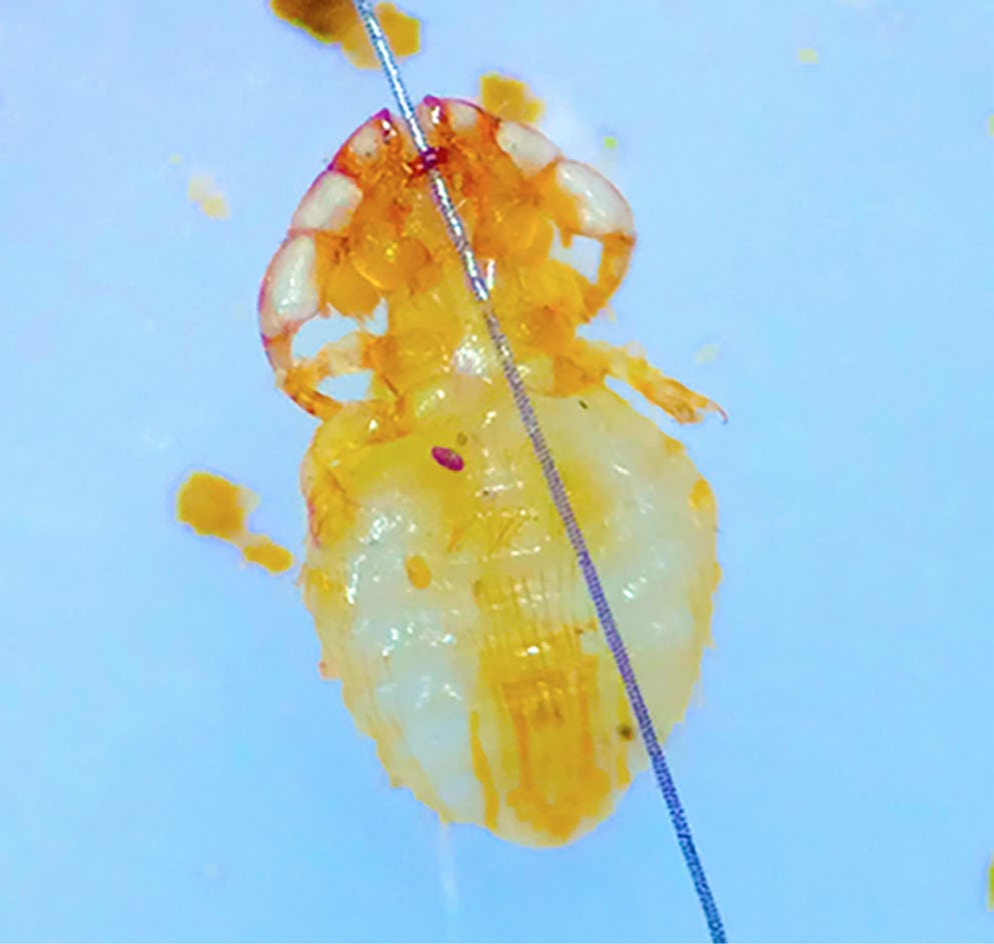
(Cover photograph) Colonizing chewing louse (*Geomydoecus aurei*, male) clasping a hair of a new host pocket gopher subspecies (*Thomomys bottae opulentus*)

Chewing lice of the genus *Geomydoecus* (Phthiraptera: Trichodectidae; Figure 1) are found only on pocket gophers. They are wingless insects that feed on skin detritus, and they spend their entire lives on their host and are highly host‐specific (Demastes, Hafner, Hafner, & Spradling, 1998; Marshall, 1981; Murray, 1957). Transmission of lice among hosts appears to require host‐to‐host contact (Timm, 1983), and the ability of a chewing louse to colonize new hosts is greatly limited by the louse's poor dispersal ability combined with the solitary nature of its host (Demastes et al., 2012; Harper, Spradling, Demastes, & Calhoun, 2015; Nadler, Hafner, Hafner, & Hafner, 1990; Nessner, Andersen, Renshaw, Giresi, & Light, 2014). As such, most colonization is from mother to offspring (Rust, 1974).

**Figure 2:**
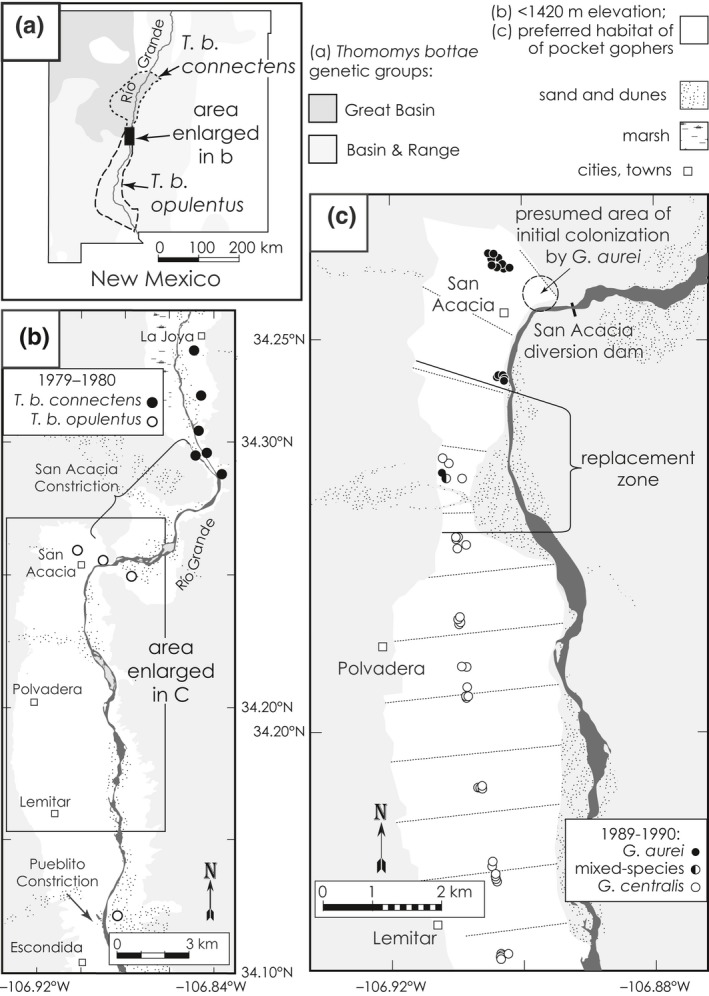
(a) Distribution of Botta's pocket gophers, *Thomomys bottae*, in New Mexico. Dark shading indicates Great Basin genetic group; light shading indicates Basin and Range genetic group (Patton & Smith, 1990). Distributions of the subspecies *T. b. connectens* and *T. b. opulentus* are indicated by dotted lines. (b) La Joya‐Escondida study area showing 1979–1980 pocket gopher samples (Smith et al., 1983). (c) Detail of the San Acacia‐Lemitar study area, showing 1989–1990 survey samples of *Geomydoecus* chewing lice and kilometers (dotted lines) along the north–south transect beginning at the San Acacia diversion dam, the area of presumed initial colonization by *G. aurei*, and the initial estimated location of the louse replacement zone

Representatives of two strongly differentiated geographic units of *T. bottae* come into contact in the Río Grande Valley of central New Mexico (Belfiore, Liu, & Moritz, 2008; Hall, 1981; Patton & Smith, 1990; Smith, 1998). Pocket gophers are largely restricted to the friable soils of the valley floor, and the broad 2–4‐km wide valley is constricted at the contact zone by an elevated ridge that forces the Río Grande through a narrow (300‐m wide), steep‐walled canyon known as the San Acacia Constriction. *Thomomys b. connectens* (representing the northern, Great Basin genetic group; Patton & Smith, 1990) occurs in the Albuquerque Basin south to La Joya (just north of the constriction), whereas *T. b. opulentus* (the southern, Basin and Range genetic group; op. cit.) occurs south of the constriction (Figure 2a). The two subspecies are more differentiated genetically than most congeneric species of other mammals (Harper et al., 2015; Patton & Yang, 1977), yet they exhibit limited hybridization with introgression at this zone (Smith, Patton, Hafner, & Hafner, 1983; sampling localities shown in Figure 2b). Subsequent studies (Demastes et al., 1998; Harper et al., 2015) demonstrated that the pocket gopher hybrid zone has not changed location since its initial discovery in 1979–1980 (Smith et al., 1983).

At the contact zone, *T. b. connectens* and *T. b. opulentus* host different species of chewing lice (*Geomydoecus aurei* and *G. centralis*, respectively) belonging to different species groups within the *G. californicus* species complex (Price & Hellenthal, 1981a). Unlike the two subspecies of pocket gophers, the two species of chewing lice that meet at this zone show fixed allelic differences, and there is no evidence of interbreeding (Demastes, 1990). Examination of dried specimens of *Geomydoecus* brushed from 15 of the *Thomomys* collected in 1979–1980 (Appendix 1) revealed *G. aurei* (the northern species of louse) near San Acacia, just south of the constriction. In other words, the northern species of louse had successfully passed through the San Acacia Constriction at least by 1979.

**Figure 3:**
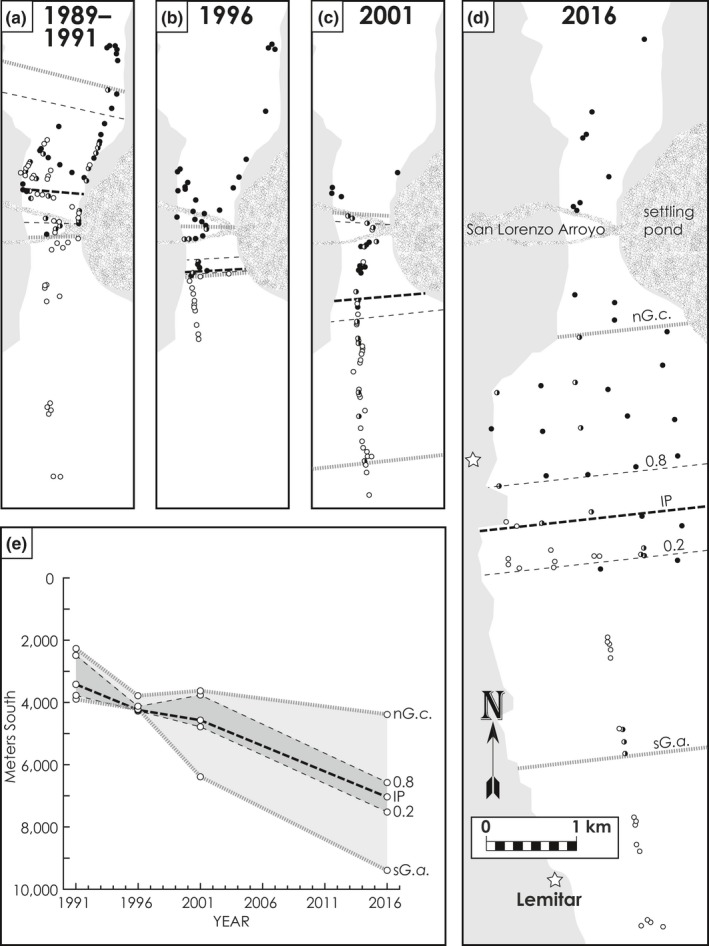
Chewing louse (*Geomydoecus*) samples collected in (a) 1989–1991, (b) 1996, (c) 2001, and (d) 2016 (all at same scale). Circles indicate individual pocket gopher hosts where filled circles = pocket gophers with only *Geomydoecus aurei*, open circles = *G. centralis* only, and half‐filled circles = mixed‐species samples. White areas indicate habitat preferred by pocket gophers; stippled pattern indicates the sandy ridges and settling basin associated with the San Lorenzo Arroyo. Locations of transition zone metrics are shown in a‐e: northernmost *G. centralis* (n*Gc*); 80% (0.8), ~50% (IP), 20% (0.2) frequencies of *G. aurei* on a pocket gopher; and southernmost *G. aurei* (s*Ga*). (e) Changes in transition zone metrics over time; light shading indicates full width of the zone (n*Gc* to s*Ga*), dark shading indicates 0.2–0.8 width of the replacement zone

While the pocket gopher hybrid zone has stabilized at the San Acacia Constriction, the chewing louse contact zone has moved continuously southward at a steady rate since 1979–1980 (Hafner et al., 1998), with the northern species of chewing louse (*G. aurei*) replacing the southern species (*G. centralis*; initial louse sampling shown in Figure 2c). Concentrated sampling in 1991 fixed the midpoint of the chewing louse replacement zone at ~6 km south of the midpoint of the pocket gopher hybrid zone (Figure 3a). Subsequent sampling 5 years later (1996) revealed that the chewing louse replacement zone had moved another 700–900 m to the south (Figure 3b). Based on the estimated annual rate of movement of the chewing louse replacement zone (140–190 m/year between 1991 and 1996), Hafner et al. (1998) concluded that initial contact between the two species of chewing louse, and possibly the most recent contact between the two subspecies of pocket gophers, had occurred after the catastrophic San Marcial floods of 1929 rather than 10,000 years ago, as suggested by Smith et al. (1983). Hafner et al. (1998) reasoned that any pocket gophers (and their chewing lice) inhabiting the narrow canyon of the San Acacia Constriction would have been extirpated by the catastrophic floods, thus obliterating any genetic signature of the pocket gopher hybrid zone remaining from previous contact. Hafner et al. (1998) concluded that the hybrid zone between the two subspecies of pocket gophers had stabilized at the San Acacia Constriction; theoretical cline models (Endler, 1977; Kohlmann & Shaw, 1991) show that partial barriers, such as the San Acacia Constriction, can attract and anchor the geographic position of clines and contact zones.

Herein, we analyze in detail data collected over nearly four decades at the San Acacia contact zone to test predictions about the dynamics of the zone. Our data, from five time periods (1979–1980, 1989–1991, 1996, 2001, and 2016), have enabled a more complete description of the rate and dynamics of the moving chewing louse replacement zone, while leading to a more precise explanation for the recent contacts between the two pocket gopher subspecies and their chewing lice. We evaluate the interactions among both pairs of taxa, employing additional metrics of the replacement zone and extrapolating back almost a century to the time of establishment of the zone. By employing these additional zone metrics, we are able to better understand the dynamics of interactions between and among the chewing lice and their pocket gopher hosts.

## 2 MATERIALS AND METHODS

### 2.1 Specimens examined

Between 1979 and 2016, 589 pocket gophers were collected for analyses of the contact zone along the Río Grande between La Joya and Escondida, Socorro Co., New Mexico; 425 of these were brushed to collect chewing lice (Hafner et al., 1998; Smith et al., 1983; this study; see Appendix 1; Figures 2 and 3). All specimens were collected under permits from the New Mexico Department of Game and Fish using methods approved by the University of Northern Iowa Institutional Animal Care and Use Committee and the American Society of Mammalogists (Sikes, 2016).

Sample size of chewing lice assayed from each pocket gopher for species assignment varied with method of analysis and location within or outside of the replacement zone: $\bar{n}$ = 45 for morphological identification of mounted specimens from throughout the study area; *n* ≤ 20 ($\bar{n}$ = 19) for allozyme analysis within the replacement zone, *n* ≤ 10 ($\bar{n}$ = 6) outside of the zone; and $\bar{n}$ = 31 for most DNA analyses except for *n* = 4 or 5 for 18 samples of chewing lice from south of the zone.

There is no evidence of interbreeding between the two louse species at this replacement zone (Demastes, 1990); thus, we were able to use simple genetic methods (described below) to assign each louse to the correct species. The chewing louse sample from each pocket gopher was scored as all *G. aurei*, mixed‐species, or all *G. centralis*. Hosts (and their chewing louse samples) were grouped into 200‐m wide bins perpendicular to a north–south transect with the zero mark set at the San Acacia diversion dam immediately south of the San Acacia Constriction (Figure 2c). For each bin, the percentage of pocket gophers hosting *G. aurei* was calculated, with individuals hosting both species (regardless of the proportions) counting as one‐half *G. aurei* and one‐half *G. centralis*. For example, a bin containing five pocket gophers, four of which hosted only *G. aurei* and one of which hosted both species, would result in a value of 90% of pocket gophers hosting *G. aurei*.

### 2.2 Genetic analyses

The pocket gopher hybrid zone initially was described based on chromosomes, allozyme electrophoresis, and morphometric analysis of cranial and pelage characters (Smith et al., 1983). Subsequent studies analyzed diagnostic allozymes (glucose‐6 phosphate dehydrogenase and mannose phosphate isomerase), mitochondrial DNA haplotypes (Demastes et al., 1998), and mitochondrial and nuclear DNA (Harper et al., 2015).

More than 6,300 individual *Geomydoecus* lice were identified to species. Specimens collected in 1991–2001 were identified to species using allozyme electrophoresis methods of Nadler and Hafner (1989) and Demastes (1990). Chewing lice from 2016 and selected individuals from 1991–2001 were identified to species using one of three molecular methods, each of which began with isolation of DNA from individual lice as described in Harper et al. (2015). Some individuals were identified to species using the Group 1 microsatellite primers and conditions published by Light, Harper, Johnson, Demastes, and Spradling (2018), which consistently amplify three loci for *G. aurei* and only two for *G. centralis* (Loci Ga3702 and Ga6020, but not Ga4103). Others were identified using a 379‐bp (base pair) region of the COI gene amplified using conditions described by Hafner et al. (1994) and either sequenced or cut with the restriction enzyme, Sau3AI (Optizyme; Thermo Fisher Scientific). Digested COI products for *G. aurei* yielded two fragments, 96 and 283 bp plus primer length, while *G. centralis* samples were not cut by Sau3AI.

### 2.3 Zone characteristics

Five‐parameter (5‐p) logistic regressions were conducted using the nplr package (version 0.1‐7) of R (version 3.3.3, R Core Team, 2013) to model the sigmoidal nature of the variables over geography. Unlike the typically employed tanh curve (Barton, 1979; Barton & Hewitt, 1981,1985; Bull & Burzacott, 2001; Szymura & Barton, 1986), the 5‐p logistic regression allows for asymmetry in the resultant sigmoidal curve. To assess position and width of the louse replacement zone for each of four time periods (1989–1991; 1996; 2001; and 2016), nine variables describing the resulting logistic curves were recorded as follows: (a) “n*Gc*,” defined as the northernmost location of the southern louse, *G. centralis*; (b) “0.8,” the point at which *G. aurei* represents 80% of the lice on gophers (the 0.8 frequency point of a conventional zone width; May, Endler, & McMurtrie, 1975); (c) “IP,” the inflection (or null) point, where *G. aurei* and *G. centralis* each represent 50% of the louse population; (s) “0.2,” the point at which *G. aurei* represents 20% of lice on gophers (the 0.2 frequency point of a conventional zone width); (e) “s*Ga*,” defined as the southernmost location of the northern louse, *G. aurei*; (f) full width of the replacement zone from northernmost *G. centralis* to southernmost *G. aurei*; (g) 0.2–0.8 width of the replacement zone; (h) goodness‐of‐fit (GOF); and (i) weighted GOF. From north to south, the replacement zone is characterized by the n*Gc*, 0.8, IP, 0.2, and s*Ga* variables. Bivariate linear regression analyses (SYSTAT 7.0; Wilkinson, 1997) were used to evaluate the statistical significance of trends in zone variables 1–5 versus geographic placement in the zone, and time of arrival of *G. aurei* at the San Acacia diversion dam was estimated by calculating *X* (year) when *Y* (distance south, in m) = 0 for significant regressions. Because it was obvious from both field observations and initial examination of the data that all five of these variables were moving southward (all show monotonic increasing trends), we employed one‐tailed *t* tests for significance of each linear trend at the *p* = 0.05 level. The null hypothesis is no significant bivariate linear association (*r* = 0), while the alternative hypothesis is that *r* > 0.

The expected proportions of single‐species and mixed‐species samples from within the 0.2–0.8 replacement zone were calculated based on Hardy–Weinberg predictions and compared to observed proportions using Chi‐square tests. Panmictic breeding among all pocket gophers and equal persistence of both species of chewing lice when living together on an individual host should follow Hardy–Weinberg predictions of proportions of single‐species and mixed‐species samples. Assuming that there are equal proportions of both species of chewing lice within the 0.2–0.8 replacement zone, the expected proportions of pure *G. aurei*, mixed‐species, and pure *G. centralis* are 1:2:1.

## 3 RESULTS

### 3.1 Movement of the chewing louse replacement zone

**Figure 4:**
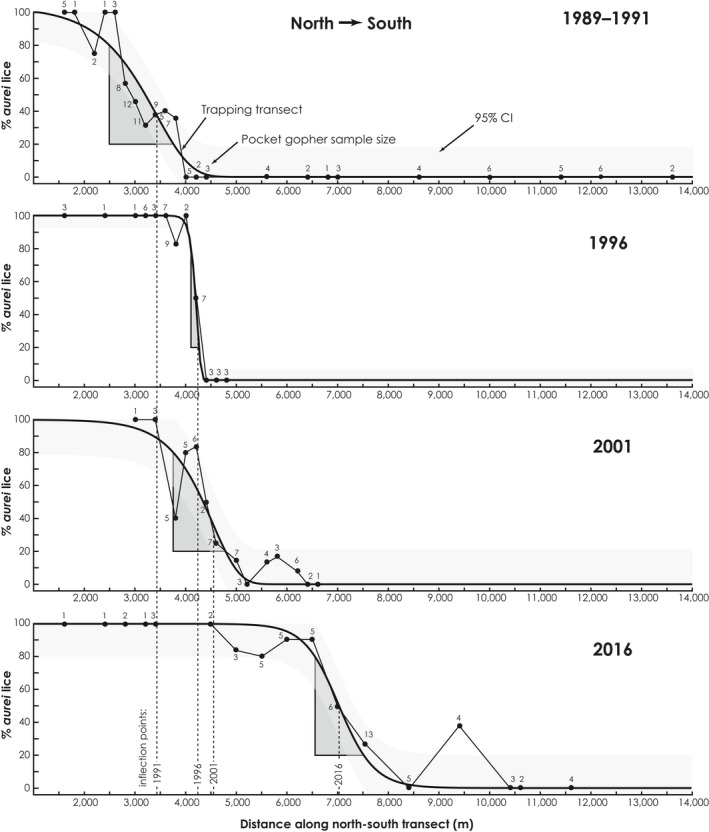
Logistic curves resulting from 5‐parameter logistic regressions of frequency of *Geomydoecus aurei* in samples of chewing lice from pocket gophers along a north–south transect (beginning at the San Acacia diversion dam) across four time intervals. Hosts and their chewing louse samples were grouped into 200‐m bins along the north–south transect. Shading indicates the 95% confidence interval about the regression, and triangles outline the conventional 0.2–0.8 cline width for each time period

**Figure 5:**
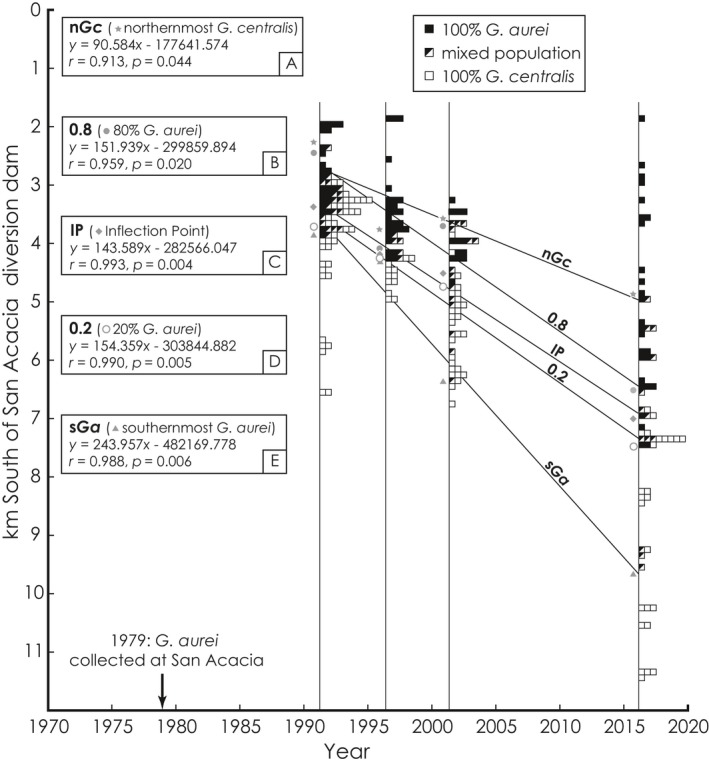
Locations of chewing louse (*Geomydoecus*) populations along a north–south transect beginning at the San Acacia diversion dam (0 km south on the *Y*‐axis) and linear regressions of zone metrics from Table 2. The *Y*‐axis is reversed to show meters south of the diversion dam

We identified samples of chewing lice from 377 pocket gophers from the study area (San Acacia to south of Lemitar, Figures 2c and 3) as pure *G. aurei*, mixed‐species samples, or pure *G. centralis*. These included 124 samples from 1989–1991 (Figure 3a), 47 from 1996 (Figure 3b), 56 from 2001 (Figure 3c), and 150 from 2016 (Figure 3d). Five separate variables (variables 1–5 of Table 1; Figure 3e) and the results of 5‐p logistic regressions for each time period (Figure 4) indicate a steady southward advance of *G. aurei*. Average rates of change in these variables for each time interval were fairly consistent, except for values from 1996 (full width and 0.2–0.8 width in Table 1). For the full 25‐year period represented by the data (1991–2016), the northernmost limit of *G. centralis* distribution has moved southward at a slower rate (84 m/year) relative to rates of movement of the 0.2–0.8 replacement zone (0.2, IP, and 0.8; 144–163 m/year, $\bar{X}$ = 152.4), whereas the rate of movement of the southernmost *G. aurei* is substantially higher (232 m/year). Regressions of variables 1–5 and the distribution of pure and mixed populations of lice along the north–south transect for each sample year (Figure 5) also indicate a relatively constant rate of southern movement for each variable throughout the 25‐year study period, with the 0.2, 0.8, and IP variables of the 0.2–0.8 replacement zone moving at a similar rate ($\bar{X}$ = 150 m/year) that is faster than that of the n*Gc* rate ($\bar{X}$ = 91 m/year) and slower than that of the s*Ga* rate ($\bar{X}$ = 244 m/year). The regressions for all five variables were significant (*p* = 0.004–0.044).

**Table 1:** Replacement zone parameters (position south of the San Acacia diversion dam, in meters) and goodness‐of‐fit values for 5‐parameter logistic curves for each time period

	Parameter	1991	1996	2001	2016
1.	Northernmost *G. centralis*	2,300	3,800	3,600	4,900
2.	0.8 (80% *G. aurei*)	2,492	4,106	3,736	6,558
3.	Inflection (null) point (50% *G. aurei*)	3,420	4,231	4,547	7,026
4.	0.2 (20% *G. aurei*)	3,760	4,266	4,786	7,516
5.	Southernmost *G. aurei*	3,900	4,300	6,400	9,700
6.	Full width (#1–#5)	1600	500	2,800	5,200
7.	0.2–0.8 width (#2–#4)	1,268	160	1,050	958
8.	Goodness‐of‐fit	9.641	9.960	9.547	9.586
9.	Weighted goodness‐of‐fit	9.989	9.999	9.985	9.985

### 3.2 Proportions of mixed‐species samples

The proportion of *G. aurei* in 15 mixed‐species samples of chewing lice with sample sizes ≥25 indicates an imbalance in proportions of the two species ($\bar{n}$ of lice per pocket gopher = 40, resulting in a confidence level of *p* = 0.025 of detecting a mixed population). We collected a total of 30 pure *G. aurei*, 30 mixed‐species samples, and 33 pure *G. centralis* from the 0.2–0.8 replacement zone during the entire study, which represents fewer mixed‐species samples than would be predicted under a process of random association and equal persistence of single‐species and mixed‐species samples (Chi‐square test, *p* = 0.039).

## 4 discussion

In this study, the dispersal rate of chewing lice across the landscape is dependent on a complicated set of nonindependent factors including pocket gopher density, rate of pocket gopher dispersal, frequency of physical contact among pocket gophers, rate of successful colonization of new host individuals by chewing lice, and rate of species replacement on a newly colonized pocket gopher. Further study is needed to reveal the nature of competitive interaction between these two species of chewing lice, be it competition for specific microhabitats on an individual pocket gopher, different reproductive rates, breeding interference, or some other interaction. Each of the metrics that we have employed to describe the replacement zone, and the rate of change in each (Tables 1 and 2; Figure 3e), is consistent across the time periods with the exception of the values from 1996. The narrowing of the replacement zone width and changes in rates of change in the 1996 period might be attributed to a narrowing of the Río Grande Valley floor that coincides with the crossing of a sandy arroyo, associated flood control dikes, and a settling pond (San Lorenzo Arroyo in Figure 3d). However, it is more likely that these departures are artifacts of the relatively narrow width of sampling along the north–south transect in 1996, and that additional mixed‐species samples, northern *G. centralis*, and southern *G. aurei* were outside of our sampling zone (Figure 5).

**Table 2:** Average annual rates of movement of parameters of the replacement zone in three time intervals and over the 25 years of the study. The estimated year of arrival of *Geomydoecus aurei* at the San Acacia diversion dam ($\bar{X}$ = 1969.5 ± 5.9 *SD*) is the average value of *X* (year) when *Y* (m south) = 0. Values involving 1996 (boldface) were likely affected by limited sampling along the north–south transect (see text)

	Time interval (year)	northernmost *G. centralis* (m)	0.8 *G. aurei* (m)	inflection point (m)	0.2 *G. aurei* (m)	southernmost *G. aurei* (m)
1991–1996	**5**	**300**	**322**	**162**	**101**	**80**
1996–2001	**5**	**−40**	**−74**	**63**	**104**	**420**
1991–2001	10	130	124	113	103	250
2001–2016	15	87	188	165	182	220
1991–2016	25	104	163	144	150	232
~1971–2016	45	98	146	156	167	213

### 4.1 Interpretations of zone metrics

Studies of contact zones usually describe only the line of contact or, at most, the width of the contact zone (Buggs, 2007). Our use of additional metrics allows more detailed description of the replacement zone, its movement, and the relative roles of both pocket gopher and chewing louse dispersal in effecting zone width and other parameters. All of our calculated rates of pocket gopher and chewing louse movement suggest that northern pocket gophers (*T. b. connectens*) bearing northern lice (*G. aurei*) from a source population near La Joya first came into contact with southern pocket gophers (*T. b. opulentus*) bearing southern lice (*G. centralis*) sometime after the catastrophic floods of 1929. Zone metrics can be used in two ways to estimate the year northern lice arrived at the San Acacia diversion dam (the zero point on the north–south transect in Figure 4). First, the southernmost *G. aurei* (s*Ga*), which marks the leading edge of southward dispersal of the species, moved 5,800 m from 1991–2016 (Table 1), or 232 m/year (Table 2). The location of s*Ga* in 2016 was at the 9,700‐m point on the north–south transect (Table 1), so extrapolating back in time using the rate estimate of 232 m/year yields 41.81 years, which means that the northern lice reached the diversion dam by around 1974 (2016 minus 42 years). Examination of the significant regressions of the zone metrics (Figure 5) provides another way to estimate time of arrival of *G. aurei* at San Acacia. Using the linear solutions in Figure 5b–e when *Y* (distance along the north–south transect) = 0, *X* (estimated year of arrival) = 1969.5 ± 5.92 *SD* (both methods include the 2016 location of s*Ga*, and so are not fully independent). Note that these two estimates (~1974 and ~1970), based solely on zone metrics, compare favorably with the observed presence of *G. aurei* in San Acacia (based on museum specimens of lice) in 1979. Also, by extrapolating back farther in time using the rate estimate of 232 m/year, it would have taken *G. aurei* 39 years to pass through the San Acacia Constriction (8,970 m by river north of the San Acacia diversion dam; Figure 2c). Thus, pocket gophers bearing *G. aurei* would have entered the constriction in 1935, soon after completion of major flood control measures (Scurlock, 1998).

Zone metrics can be used to investigate aspects of the zone not normally associated with traditional studies of contact zones. For example, the question, “How long does it take one parasite species to replace another within a population of hosts?” can be addressed using zone metrics alone. Width of the 0.2–0.8 replacement zone reflects the speed with which northern lice invade and replace southern lice on a local population of pocket gophers, and so measures diffusion dispersal from the perspective of the chewing louse. Zone width narrows as frequency of invasion (contact between the asocial pocket gophers, which allows transfer of northern lice to pocket gophers bearing only southern lice) and rate of replacement of southern lice by northern lice increase. Zone width calculated using the conventional 0.2–0.8 method (May et al., 1975) is relatively constant at ~1 km (1,092 ± 159.2 *SD* m) for the periods 1991, 2001, and 2016. At a rate of 150 m/year, the 0.2–0.8 replacement zone takes nearly 7 years to move 1 km. In other words, it takes ~7 years for *G. aurei* to grow from 20% of the overall louse population at a locality to 80% of the population. The additional time required for *G. aurei* to expand from 80% to 100% of the population is estimated below using a different zone metric.

Because louse dispersal is directly dependent on gopher dispersal, the rate of southward movement of the 0.2–0.8 replacement zone reflects the average annual dispersal distance of pocket gophers. Our estimated rate of mean annual dispersal distance for pocket gophers based solely on zone metrics ($\bar{X}$ of regression slopes of 0.2, IP, and 0.8 parameters, Figure 5b–d) is 149.96 ± 5.65 *SD* m/year. This estimate, based on 25 years of monitoring over a distance of 10 km, is higher than those reported in the literature based on direct observation, usually trapping data (e.g., 62 m/year, Howard & Childs, 1959; 78 m/year, Vaughan, 1963; 117 m/year, Daly & Patton, 1990), but it is widely acknowledged that annual dispersal distances are probably site specific and depend on resource availability, population density, and potentially many other ecological factors that influence animal movement.

The southernmost occurrence of *G. aurei* (s*Ga*) measures jump dispersal (Lomolino, Riddle, & Brown, 2006) from the perspective of the chewing louse, as it marks the maximum southward dispersal distance of a successful pocket gopher colonist bearing *G. aurei* lice. Such long‐distance dispersal starts a new colony of *G. aurei* deep within the range of *G. centralis*, much as wind‐borne embers can start a “jump fire” far beyond the main body of the original fire. Our estimate of maximum annual dispersal distance for pocket gophers based solely on zone metrics (s*Ga*; $\bar{X}$ = 232 m/year for the period 1991–2016, Table 2; regression slope of 239 m/year, Figure 5e) compares favorably with the maximum dispersal distances reported in the literature based on direct observation (e.g., 274 m/year, Howard & Childs, 1959; 122 m/year, Vaughan, 1963; 300 m/year, Daly & Patton, 1990; the mean of these three estimates is 232 m/year).

The northernmost occurrence of *G. centralis* (n*Gc*) lags behind the 0.2–0.8 replacement zone, reflecting the additional time it takes for *G. aurei* to fully replace *G. centralis* (i.e., expand from 80% to 100% of the louse population) in a local population of hosts following initial colonization. The regression for n*Gc* based on the 1991–2016 data (Figure 5a) indicates southward movement of the n*Gc* at 91 m/year. The full width of the replacement zone (from n*Gc* to s*Ga*) continually expands over time because of differences in the average rates of southern movement of n*Gc* (91 m/year), the 0.2–0.8 replacement zone (150 m/year; the average annual southward dispersal distance of the hosts), and s*Ga* (the maximum annual southward dispersal distance of the hosts; 244 m/year).

Based on transect data from 2016 (Table 1) and rates of movement estimated solely from zone metrics, a stationary observer monitoring louse populations in late 2015 at a point 9,700 m south of the San Acacia diversion dam (the 2016 location of the southernmost *G. aurei*) would have witnessed the arrival of the first northern lice (*G. aurei* “jump dispersers”) in a louse population that had previously been 100% southern lice (*G. centralis*). Nearly, 15 years would pass (9,700 − 7,516 = 2,184 m at 150 m/year) before *G. aurei* louse populations in the vicinity of the observer were 20% northern lice, and another 6.4 years would pass (958 m at 150 m/year) before *G. aurei* expands to 80% of the louse population. Finally, 27 additional years would pass (2,459 m at 91 m/year, for a total of 48 years, the year 2064) before all lice in the vicinity of the observer are *G. aurei*. Forty‐eight years for one parasite to completely replace another seems slow from the human perspective, but it is a mere wink of the eye in terms of ecological, much less, geological time.

New knowledge about this contact zone, including rate of zone movement, average and maximum dispersal distance of gophers (hence their lice), and rate of replacement of one parasite by another, would not have been discovered in a traditional contact zone study involving a single (or a few) localities at a single point in time. The common assumption that contact zones are geographically stationary belies the empirical fact that species’ ranges change frequently, often as a result of replacement of one species by another at zones of competitive parapatry.

### 4.2 Geographic and temporal context of the contact zone

Contact between these two subspecies of pocket gophers occurred at a biogeographically unique location along the Río Grande at a singular time in the history of the river's flood regime. In a larger geographic context, the San Acacia Constriction marks the meeting place between the Great Basin and Chihuahuan Desert biomes (Bailey, 1913; Küchler, 1985), including the two major genetic groups of *Thomomys bottae* represented by *T. b. connectens* and *T. b. opulentus* (Great Basin and Basin and Range clades, respectively, as defined by Patton & Smith, 1990; Figure 2a). In a larger temporal context, contact between the Great Basin and Chihuahuan Desert clades of *T. bottae* occurred following a major, human‐induced change in the natural flood cycle of the Río Grande. Historically, the Río Grande Valley experienced regular and often extreme spring flooding from snowmelt and summer‐monsoon flash flooding (Scurlock, 1998), and the bosque ecosystem evolved in this annual flood regime (Cartron, Lightfoot, Mygatt, Brantley, & Lowrey, 2008). These floods must have regularly scoured pocket gophers from the San Acacia Constriction and temporarily reduced population density elsewhere along the river. The catastrophic San Marcial floods of 1929 occurred prior to completion of flood control measures (Harden, 2006; Lee, 1907; Patterson, 1965; Scurlock, 1998; Sodrensen & Linford, 1967). Eventually, however, flood control and diversion dams, as well as systems of irrigation ditches and local channelization of the Río Grande, moderated both flooding and drought periods of the river and constrained the river to a narrower channel, permitting year‐around agriculture of the Río Grande floodplain, particularly extensive growing of alfalfa. These same flood control measures also allowed the two *Thomomys bottae* subspecies of pocket gophers to come into contact. Instead of the major floods of the 1920s and 1930s eradicating any evidence of previous contact (as argued by Hafner et al., 1998), we contend that contact prior to the establishment of flood control measures was prevented by the historical flood regime of the Río Grande.

The southern louse species in this study, *G. centralis*, represents an isolated and geographically restricted population of a species that is widespread across the Great Basin (Price & Hellenthal, 1981a). Prior to ~1971, this isolated population of *G. centralis* hosted by *T. b. opulentus* ranged from San Acacia southward 70 km to San Marcial, but has surrendered nearly 15% of its estimated 17,500 ha distribution to *G. aurei* during the course of our study. At the current rate of replacement and zone movement, *G. aurei* will reach San Marcial in ~250 years, and, unless conditions change, will completely extirpate this population of *G. centralis* in ~650 years.

### 4.3 Congruence between pocket gopher and chewing louse distributions

The association between pocket gophers chewing lice is literally a textbook example of cospeciation (Coyne & Orr, 2004; Esch & Fernández, 1993; Futuyma, 2005; Lehane, 2005; Lomolino et al., 2006; Morris, 2013; Nardon, 2000; Noble, Noble, Schad, & MacInnes, 1989; Page & Holmes, 1998; Price, Denno, Eubanks, Finke, & Kaplan, 2011; Ridley, 2004; Taubes, 2001; Willmer, Stone, & Johnston, 2005). As such, we expect—and usually observe—congruence between the geographic distribution of a gopher taxon and the taxon of louse parasitizing that gopher. Hellenthal and Price (1984) found a general correspondence between the distribution of the then four recognized species of pocket gophers of the *Thomomys* subgenus *Megascapheus* and 8 species complexes of their chewing lice (comprising 29 species of *Geomydoecus*). Advances in the understanding of evolutionary relationships within *T. bottae* (Belfiore et al., 2008; Patton & Smith, 1990; Smith, 1998) and *T. umbrinus* (now itself composed of four species; Hafner, Hafner, Patton, & Smith, 1987; Hafner, Gates, Mathis, Demastes, & Hafner, 2011; Mathis, Hafner, Hafner, & Demastes, 2013a; Mathis, Hafner, Hafner, & Demastes, 2013b; Mathis, Hafner, & Hafner, 2014) permit a more informed evaluation of general patterns of distributional relationships within these pocket gophers and their chewing lice.

To date, the distribution of species of *Geomydoecus* lice relative to their hosts has been evaluated at one other hybrid zone of *Thomomys*. Patton, Smith, Price, and Hellenthal (1984) studied a hybrid zone between *T. bottae* (hosting *G. shastensis*) and *T. townsendii* (hosting *G. idahoensis*) in northeastern California that had been described previously by Thaeler (1968). Patton et al. (1984) concluded that the pocket gopher hybrid zone had been stationary for at least 25 years without introgressive hybridization, and that the contact zone between their nonhybridizing chewing louse species was concordant with the narrow host hybrid zone.

This contact zone, a zone of competitive parapatry (sensu Haffer, 1986; Bull, 1991) from the louse's perspective, is likely to be only one of many such zones among the mosaic of 29 species of chewing lice hosted by the seven species of *Thomomys* in the subgenus *Megascapheus* that cover most of the western United States and northern Mexico. Comparison of the distributions of *Geomydoecus* species (Hellenthal & Price, 1984) and *Thomomys* (subgenus *Megascapheus*) species and genetic groups (Mathis et al., 2014; Patton & Smith, 1981,1990; Smith, 1998) indicate multiple instances of apparent range overlap that may have resulted from past host switching or may represent current zones of competitive parapatry between species of chewing lice (summarized in Table 3). Published records of louse localities (Hellenthal & Price, 1980; Price & Hellenthal, 1979, 1980, 1981a, 1981b) already reveal 35 specific sites where multiple species of lice have been collected from a single population of pocket gophers (Table 3). These localities of sympatry not only confirm many of the apparent instances of range overlap, but may represent geographically stable zones of competitive parapatry or moving zones of species replacement. Just as “movement of hybrid zones in the present and recent past could be a widespread phenomenon” (Buggs, 2007:307), untested assumptions of stasis of species contact (without hybridization) may mask a more widespread existence of ongoing species replacement following recent environmental perturbations or ongoing climatic change. Thus, the localities of sympatry may provide additional opportunities to evaluate the spatio‐temporal dynamics of both the louse species and their pocket gopher hosts over long periods of time, as well as the nature of species interactions in the zone of competitive parapatry. Such zones may signal past or ongoing human disturbances (as in this study) or other ecological change not previously detected. Our demonstration that zone metrics can be used to estimate natural history parameters of the species involved is a relatively new finding that portends well for future contact zone studies in which preserved specimens are not available to verify dating estimates based exclusively on zone metrics and empirical studies of dispersal distance are not available to corroborate distance estimates, again based solely on zone metrics. Comparison of multiple zones of parapatry within *Geomydoecus* may reveal common patterns among zones of parapatry in *Geomydoecus* that may better inform future studies of zones of competitive parapatry in other taxa.

**Table 3:** Geographic range overlap between species and genetic groups of *Thomomys* (subgenus *Megascapheus*) pocket gophers and species of *Geomydoecus* chewing lice: **█** indicates the major host of each chewing louse species; "s" indicates alternate louse species associations suggested by range overlap; "**X**" indicates alternate louse species associations that are confirmed by one or more known localities (Price & Hellenthal, 1979, 1980, 1981a, 1981b; Hellenthal & Price, 1980)

Host	*Geomydoecus* sp.	*Tbu*	*Tt*	NC	GB	CC	BR	BC	SON	*Tui*	*Tug*	*Tud*	*Tuu*	*Tsc*	*Tss*	*Tap*	*Taa*
*T. bulbivorus* (*Tbu*)	*oregonus*	**█**															
*T. townsendii* (*Tt)*	*idahoensis*		**█**	**X**	**X**												
*T. bottae* genetic groups:																	
Northern California (NC)	*shastensis*		**X**	**█**	**X**	**X**											
Great Basin (GB)	*centralis*		**X**		**█**		**X**		**X**	**X**							
	*aurei*				**█**		**X**										
Central California (CC)	*californicus*				**X**	**█**											
	*angularis*					**█**											
	*hueyi*					**█**											
Basin & Range (BR)	*subcalifornicus*				**X**	s	**█**		**X**	**X**							
	*limitaris*						**█**										
	*actuosis*						**█**										
	*fulvi*						**█**										
	*albati*						**█**										
	*guadalupensis*						**█**										
Baja California (BC)	*bajaiensis*					s		**█**									
	*clausonae*							**█**									
Sonora (SON)	(no unique sp.)																
*T. u. intermedius* (*Tui*)	*crovelloi*									**█**							
*T. u. goldmani* (*Tug*)	*warmanae*									**X**	**█**		s	s			
*T. u. durangae* (*Tud*)	(no unique sp.)																
*T. u. umbrinus* (*Tuu*)	*welleri*									**X**	s	s	**█**	s	s		
	*tolucae*												**█**				
*T. s. chihuahuae* (*Tsc*)	*chihuahuae*						**X**			**X**	s			**█**	s		
*T. s. sheldoni* (*Tss*)	*pattoni*														**█**		
*T. a. parviceps* (*Tap*)	*sinaloae*															**█**	
	*umbrini*															**█**	
*T. a. atrovarius* (*Taa*)	*musculi*																**█**
	*nayaritensis*																**█**
	*jaliscoensis*																**█**
	*extimi*																**█**
	*cliftoni*																**█**
*T. nayaritensis*	(none identified)																

## CONFLICT OF INTEREST

None declared.

## AUTHOR CONTRIBUTION

DJH and MSH conceived the idea for this synthesis and wrote the article; JWD and TAS prepared and analyzed the data; all authors participated in revising the manuscript; JWD, TAS, and JEL secured funding for the project.

## Data Availability

Chewing louse microsatellite genotypes and R‐scripts are available in Dryad, https://doi.org/10.5061/dryad.9sv4q00. Parasite voucher specimens and frozen tissue samples from pocket gophers are stored at −80°C at the University of Northern Iowa and at Louisiana State University. Pocket gopher voucher specimens are housed at the Louisiana State University Museum of Natural Science (LSUMZ), the Museum of Southwestern Biology, University of New Mexico (MSB), Museum of Vertebrate Zoology, University of California (MVZ), or the Biodiversity Research and Teaching Collections at Texas A&M University (BRTC). Current and future research directions of the team may be found at https://louselab.uni.edu/homepage.

## APPENDIX 1

### SPECIMENS EXAMINED

Specimens of *Thomomys bottae* (*n* = 589) included in this study are housed in either the Louisiana State Museum of Natural Science (LSUMZ; specimens from 1989–1996), Museum of Southwestern Biology, University of New Mexico (MSB; 1990–2001), Museum of Vertebrate Zoology, University of California (MVZ; 1979–1989), or the Texas A&M University Biodiversity and Research Teaching Collections (TCWC; 2011–2016). Specimens of *Geomydoecus* included in this study (populations from 425 hosts; approximately 6,300 specimens examined for this study) are maintained at −80°C at the University of Northern Iowa. In addition, dried specimens of *Geomydoecus* brushed from 15 of the *Thomomys* collected in 1979–1980, mounted on slides and identified to species, are housed in the Insect Collection of the University of Minnesota (UMSP). Samples of *Thomomys* are plotted in Figure 2; populations of *Geomydoecus* are plotted in Figures 2 and 3. Sample sizes for each analysis are indicated (LM = mounted specimens from UMSP; LA = louse allozymes; LS = louse sequences).

*Thomomys bottae connectens* (*n* = 152): New Mexico: Socorro Co.; **1979–1980** (*n* = 125, used in analyses of morphology [*n* = 57], pelage color [*n* = 69], allozymes [*n* = 112], and chromosomes [*n* = 25]; only grouped localities listed in Smith et al., 1983); 0.5 mi. S La Joya (1979, *n* = 12, LM = 2; MVZ 159,103–158,114); 2 mi. S La Joya (1980, *n* = 22, LM = 2; MVZ 158,568–158,589); 3 mi. S La Joya (1980, *n* = 13, LM = 2; MVZ 158,590–158,602); 3.5 mi. S La Joya (1980, *n* = 23, LM = 1; MVZ 158,603–158,625); 3.5 mi. S La Joya, W side Río Grande (1980, *n* = 39; MVZ 158,626–158,664); 4.0 mi. S La Joya (1980, *n* = 16, LM = 2; MVZ 158,665–158,678, 198,540–198,541). **1989–1993** (*n* = 16; Hafner et al., 1998); 1 mi. S La Joya (1992, *n* = 10, LS = 4; LSUMZ 30,737, 30,740, 30,742–30,744, 30,785–30,786, 33,915, 36,134, 36,213); 3.5 mi. S La Joya, W side Río Grande (1989, *n* = 6, LM = 1, LA = 6; LSUMZ 29,545–29550); **2011–2016** (*n* = 11; this paper); 0.9 mi. S, 0.1 mi. W La Joya (2011, *n* = 5, LS = 4; TCWC 64,971–64,975); S of La Joya (2016, *n* = 6, LS = 5; TCWC 64,297–64,302).

*Thomomys bottae opulentus* (*n* = 437): New Mexico: Socorro Co.; **1979–1980** (*n* = 39, used in analyses of morphology [*n* = 19], pelage color [*n* = 20], allozymes [*n* = 39], and chromosomes [*n* = 2]; grouped localities listed in Smith et al., 1983); 1 mi. N San Acacia Dam (1980, *n* = 2, LM = 2; MVZ 158,679–158,680); San Acacia (1979, *n* = 10, LM = 2; MVZ 158,061–158,070); San Acacia Dam, E side Río Grande (1980, *n* = 19, LM = 3; MVZ 158,681–158,699); Escondida, E side Río Grande (1980, *n* = 8; MVZ 158,700–158,707). **1989–1993** (*n* = 145; Hafner et al., 1998); San Acacia (1989, *n = *9, LA = 9; LSUMZ 29,509–29,510, 29,551–29,557), 0.7 mi. S, 0.2 mi. E San Acacia (1990, *n* = 5, LA = 5, LS = 1; MSB 287,497–287,499, 287,571–287,572); 1.5 mi. S San Acacia (1991, *n* = 20, LA = 20, LS = 5; LSUMZ 30,714, 30,732, 30,787–30,788, 30,828, 30,830, 30,865, 30,867, 30,870–30,872, 30,874–30,875, 30,904–30,905, 30,907, 30,909, 30,913, 30,918–30,919; 1992, *n* = 4, LA = 4, LS = 1; LSUMZ 30,928–30,931); 2 mi. N, 0.5 mi. E Polvadera (1990, *n* = 5, LA = 5; MSB 287,478–287,480, 287,495–287,496; 1991, *n* = 21, LA = 21, LS = 1; LSUMZ 30,731, 30,789, 30,831, 30,834–30,835, 30,861–30,862, 30,869, 30,873, 30,876, 30,902–30,903, 30,910–30,912, 30,915–30,917, 30,920–30,922; 1992, *n* = 2, LA = 2, LS = 1; LSUMZ 30,924, 30,932); 1.7 mi. N, 0.5 mi. E Polvadera (1991, *n* = 12, LA = 12; LSUMZ 30,734, 30,784, 30,790, 30,829, 30,833, 30,860, 30,866, 30,868, 30,901, 30,906, 30,908, 30,914); 1.3 mi. N, 0.3 mi. E Polvadera (1990, *n* = 5, LA = 5; MSB 287,518, 287,532–287,535); 0.8 mi. NE Polvadera (1990, *n* = 4, LA = 4; MSB 287,476, 287,624–287,626); 0.6 mi. S, 0.8 mi. E Polvadera (1990, *n* = 6, LA = 6; MSB 287,590–287,592, 287,595, 287,609–287,610); 1.8 mi. SE Polvadera (1990, *n* = 4, LA = 4; MSB 287,477, 287,621–287,623); 2.0 mi. S, 1.0 mi. E Polvadera (1992, *n* = 8, LA = 8; LSUMZ 33,393, 33,398, 33,876–33,879, 33,911–33,912); 0.7 mi. N, 0.5 mi. E Lemitar (1990, *n* = 6, LA = 6; MSB 287,513–287,516, 287,536–287,537); 0.5 mi. E Lemitar (1990, *n* = 5, LA = 5; MSB 287,604–287,608); 0.7 mi. S, 1.0 mi. E Lemitar (1990, *n* = 6, LA = 6; MSB 287,573–287,576, 287,593–287,594); 1.5 mi. S, 0.9 mi. E Lemitar (1990, *n* = 2, LA = 2; MSB 287,517, 287,552); 1.0 mi. N Escondida (1992, *n* = 12; LSUMZ 30,725–30,726, 30,729–30,730, 30,738–30,739, 30,741, 30,923, 30,925, 33,914, 34,032, 35,242); Escondida (1990, *n* = 3, LA = 3; MSB 287,589, 287,602–287,603; 1992, *n* = 5, LA = 5; LSUMZ 34,012, 35,219, MSB 272,925–272,927; 1993, *n* = 1, LA = 1; LSUMZ 34,337). **1996** (*n = *47; Hafner et al., 1998); 1.5 mi. S San Acacia (*n* = 12, LA = 12, LS = 2; LSUMZ 35,999–36,000, 36,003, 36,007–36,008, 36,010, 36,012, 36,015–36,019); 2 mi. N, 0.5 mi. E Polvadera (*n* = 35, LA = 35, LS = 3; LSUMZ 35,974–35,998, 36,001–36,002, 36,004–36,006, 36,009, 36,011, 36,013–36,014, 36,020). **2001** (*n* = 56, Hafner et al., 1998); 1.5 mi. S San Acacia (*n* = 6, LA = 6, LS = 2; MSB 273,860, 287,630, 287,873–287,874, 287,922, 287,945); 2 mi. N, 0.5 mi. E Polvadera (*n* = 50, LA = 50, LS = 2; MSB 287,553–287,557, 287,611–287,614, 287,627–287,629, 287,631–287,632, 287,824–287,829, 287,848–287,853, 287,872, 287,875–287,877, 287,896–287,901, 287,920–287,921, 287,923–287,925, 287,943–287,944, 287,946–287,948, 287,968–287,971). **2011–2016** (*n* = 150; this paper); vicinity of San Acacia (2016, *n* = 16, LS = 5; TCWC 64,303–64,318); vicinity of Polvadera (2016, *n* = 115, LS = 47; TCWC 64,246–64,271, 64,284–64,296, 64,319–64,394); vicinity of Lemitar (2016, *n* = 17, LS = 15; TCWC 64,241–64,245, 64,272–64,283); 1.1 mi. S, 0.75 E Lemitar (2011, *n* = 2; TCWC 64,969–64,970).
